# Distinct Carbonate Lithologies in Jezero Crater, Mars

**DOI:** 10.1029/2020GL092365

**Published:** 2021-05-06

**Authors:** Allison M. Zastrow, Timothy D. Glotch

**Affiliations:** ^1^ Stony Brook University Stony Brook NY USA

**Keywords:** Martian geology, remote sensing, spectral analysis

## Abstract

Jezero crater is the landing site for the Mars 2020 Perseverance rover. The Noachian‐aged crater has undergone several periods of fluvial and lacustrine activity and phyllosilicate‐ and carbonate‐bearing rocks were formed and emplaced as a result. It also contains a portion of the regional Nili Fossae olivine‐carbonate unit. In this work, we performed spectral mixture analysis of visible/near‐infrared hyperspectral imagery over Jezero. We modeled carbonate abundances up to ∼35% and identified three distinct units containing different carbonate phases. Our work also shows that the olivine in Jezero is predominantly restricted to aeolian deposits overlying the carbonate rocks. The diversity of carbonate phases in Jezero points to multiple periods of carbonate formation under varying conditions.

## Key Points

- Unmixing of near‐infrared spectra in Jezero crater suggests carbonate abundances up to ∼35% per pixel
- Strong olivine spectral signatures are correlated to sand deposits overlying light‐toned carbonate‐bearing rocks
- Fe‐carbonate is the dominant phase throughout the study area; Mg‐carbonate is present along the Jezero paleolake rim

## 1 Introduction

**Figure 1:**
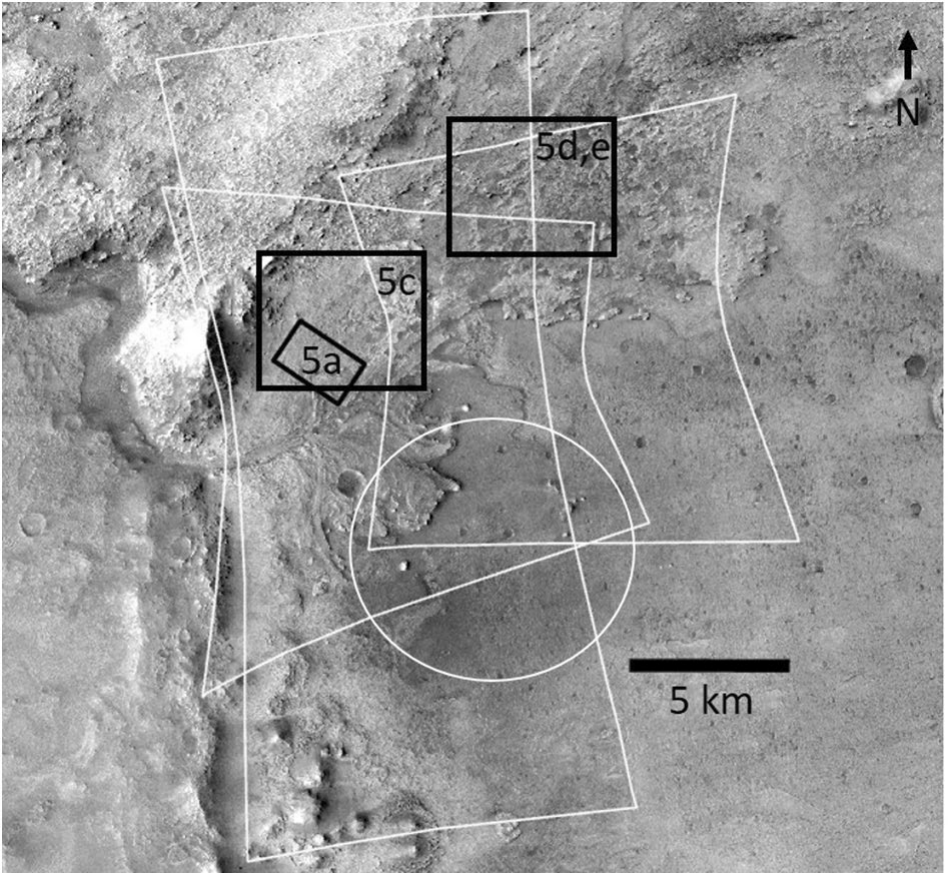
Summary of imagery and study area in this work. Yellow polygons outline the three CRISM images analyzed: FRT000047A3, FRT00005C5E, and HRL00040FF. Base image is a Context Imager (CTX) mosaic. CRISM, Compact Reconnaissance Imaging Spectrometer for Mars.

Jezero crater is the landing site for the Mars 2020 Perseverance rover (Farley et al., 2020; Williford et al., 2018). The crater was formed in the late‐Noachian to mid‐Noachian and has undergone significant periods of fluvial and lacustrine activity, resulting in the formation of phyllosilicate‐bearing and carbonate‐bearing rocks, as well as its visually striking western delta deposit (Figure 1). It also contains an exposure of the regional Nili Fossae olivine‐carbonate unit, which has long been of interest to researchers due to its status as the largest carbonate‐bearing unit identified on Mars' surface (Ehlmann et al., 2008). Previous studies (e.g., Edwards & Ehlmann, 2015; Salvatore et al., 2018) have estimated the abundances of carbonate and olivine in this regional unit, but not in Jezero at the scale of our study.

Previous studies have identified three carbonate‐bearing units in Jezero. Goudge et al. (2015) first identified two units: the Light‐Toned Floor and the Mottled Terrain, which they determined were likely stratigraphically equivalent. Horgan et al. (2020) identified a distinct unit within the Mottled Terrain, which they termed the Marginal Carbonates. Here, we discuss the similarities and differences between the Mottled Terrain and Marginal Carbonates and identify a third carbonate‐bearing unit intermixed with the olivine‐rich sands found in Jezero.

In this work, we perform spectral mixture analysis of Mars Reconnaissance Orbiter (MRO) Compact Reconnaissance Imaging Spectrometer for Mars (CRISM) visible/near‐infrared (VNIR) hyperspectral imagery over the Jezero deltaic region to estimate abundances of carbonate, olivine, phyllosilicates, and other minerals in the region and to constrain the origins of the lithologies and increase our understanding of how the rocks have been altered through time. Analysis of CRISM imagery is typically limited to feature identification, although recent advances have been made in quantitative unmixing of VNIR hyperspectral imagery using radiative transfer theory (e.g., Edwards & Ehlmann, 2015; Lapotre et al., 2017; Liu et al., 2016, 2012; Michalski et al., 2019) and advanced denoising techniques (Itoh & Parente, 2021). By unmixing single CRISM pixels, we can better understand both the composition of geologic units and their spatial extent. Our model incorporates several different mineral phases that can help tease out differences in mineral assemblages and subsequent potential formation mechanisms. In particular, the use of three carbonate endmembers—calcite, magnesite, and siderite—has resulted in the identification of three carbonate‐rich units in Jezero.

## 2 Data and Methods

We present an analysis of three VNIR hyperspectral image cubes acquired by CRISM (Murchie et al., 2007). Our model uses the calibrated TRR3 L detector data between 1.0 and 2.5 μm. Dust and ice opacities for each image vary based on the atmospheric conditions at the time the image was acquired and are closely related to unmixing model uncertainty in that increased atmospheric dust and ice can obscure other mineral signatures and alter modeled abundances (Zastrow & Glotch, 2018). Opacity values and image resolutions are provided in Table S1. Due to the overlap of the CRISM images, abundance maps presented in this paper will show the images in the following order (from north to south): FRT000047A3, HRL000040FF, FRT00005C5E.

In this study, CRISM data processing and analysis are essentially a two‐step process: (1) atmospheric correction and calculation of surface single scattering albedo (SSA) using a radiative transfer model and (2) spectral mixture analysis of surface single scattering albedo using a linear least squares model.

### 2.1 Atmospheric Correction

To quantitatively correct for the atmospheric contribution to CRISM I/F data (the radiance detected by the instrument divided by the solar irradiance divided by π), we use a radiative transfer model developed at Washington University in St. Louis and described by Liu et al. (2016). A detailed description is provided in the supplementary material (Text S1).

### 2.2 Nonnegative Linear Least Squares Unmixing Model

For a library of minerals, optical constants are used to build a spectral library of SSAs at various grain sizes. These mineral SSAs are used as endmembers and combined linearly to model the surface SSAs output from DISORT. The model used in this work is the nonnegative linear least squares unmixing model (NNLS, Lawson & Hanson, 1995; Rogers & Aharonson, 2008), which has been used to unmix CRISM hyperspectral images by Liu et al. (2012, 2016) and Michalski et al. (2019). In this model, the least squares fit is constrained to include only positive abundances, as negative mineral abundances have no physical meaning.

#### 2.2.1 Spectral Library

The mineral library used in the Jezero model consists of five phyllosilicates (Mg‐serpentine, montmorillonite, nontronite, saponite, and talc), three carbonates (calcite, magnesite, and siderite), four olivines (Fo_0_, Fo_40_, Fo_70_, and Fo_100_), two feldspars (anorthite and labradorite), four pyroxenes (diopside, enstatite (two samples), and augite), opal, ferrihydrite, and modeled Martian dust (Wolff et al., 2009). No sulfates were used in this model. Various grain sizes (Table S2) were chosen for each mineral group based on intensive testing which led to the combinations with the lowest root‐mean‐squared errors (RMS). We note here that out of necessity we use mineral endmembers of distinct compositions, while it is possible that the model represents mineral solid solutions with different mixing proportions of these endmembers (e.g., magnesite and calcite modeled together may actually represent a mixed cation carbonate).

## 3 Modeling Results

For each CRISM image, we ran a series of models with various combinations of endmember minerals and compared their RMS errors and modeled spectra to determine the set of endmembers that best fit the measured CRISM spectra. The best‐fit models are presented here. In addition to these “baseline” models, we also include below some discussion of the models in which the Fe‐carbonate endmember was removed, as it particularly impacts the modeled olivine distribution as compared to previous works. Additional details on the spectral library and model selection are provided in the supplementary material (Text S2).

The three modeled images show good agreement in overlapping areas, particularly north of the crater's inlet channel (where the majority of our analysis is focused). Olivine is slightly higher in HRL000040FF than the other two images, but the other groups have very similar values. We use the error associated with the dust and ice opacities to determine model error, which is largely driven by the atmospheric conditions at the time of image acquisition. This error varies by mineral group, as shown in Figure S4. The phyllosilicate and dust endmembers present the largest variation in modeled abundances between the lower and upper opacity bounds and thus their reported values have the highest uncertainty. The carbonate, opal, and olivine groups have the lowest uncertainty.

**Figure 2:**
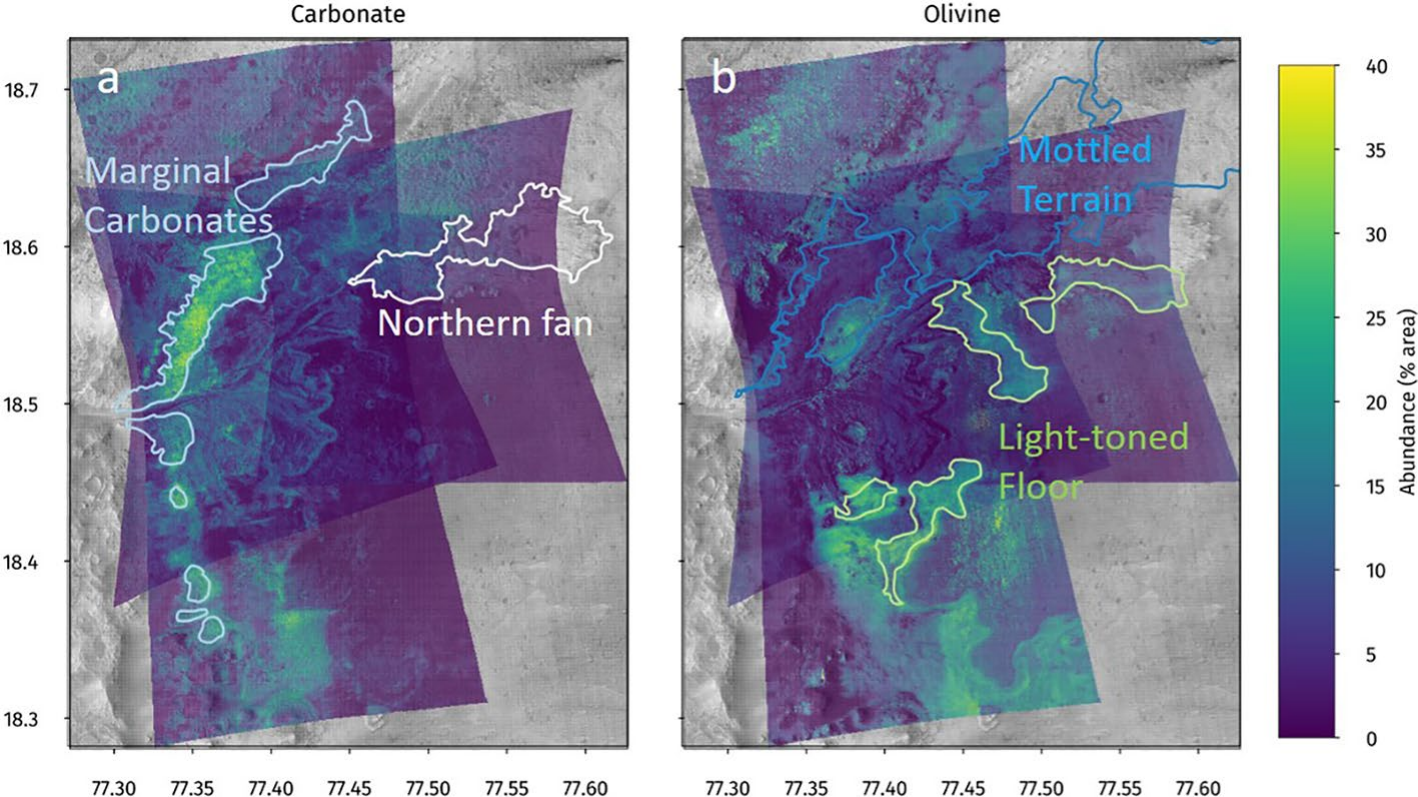
Cumulative abundance maps for (a) carbonate and (b) olivine. Outlines of the northern fan, Mottled Terrain, and the Light‐Toned Floor are from Goudge et al. (2015); the Marginal Carbonates are outlined based on Horgan et al. (2020).

For each image, the unmixing model produces cumulative abundance values for all three endmember carbonates up to ∼35% per pixel. The high‐carbonate pixels (roughly ranging from 25% to 35%) are preferentially located in the Marginal Carbonates unit of Horgan et al. (2020), as shown in Figure 2a. There are also elevated carbonate abundances located along the edges of the delta. The Mottled Terrain contains lower, but still nonzero, modeled carbonate abundances.

Cumulative olivine abundance values in some regions reach up to ∼40% per pixel. The highest olivine abundances are found inside the crater, predominately covering the Light‐Toned Floor to the south and east of the delta. The maps also indicate elevated olivine abundances along the inner‐rim of the crater, as shown in Figure 2b.

Cumulative modeled phyllosilicate abundances reach up to ∼80% per pixel in some areas (Figure 2c). The highest abundances fall largely along the inner‐rim of the crater (the same area as the elevated olivine abundances noted above), in the Mottled Terrain, and on the crater floor. As discussed in Section 4.3, we believe these abundances to be unrealistically high.

Feldspars (largely modeled as anorthite, up to ∼40%) and pyroxenes (augite and diopside, up to ∼15–20%) are also present in higher modeled abundances on the crater floor than elsewhere. The single opal endmember has modeled abundances up to ∼15–20%, covering the Mottled Terrain and the Light‐Toned Floor unit. Modeled dust abundances do not appear to be correlated with any specific landforms, and the model does not use ferrihydrite at all.

**Figure 3:**
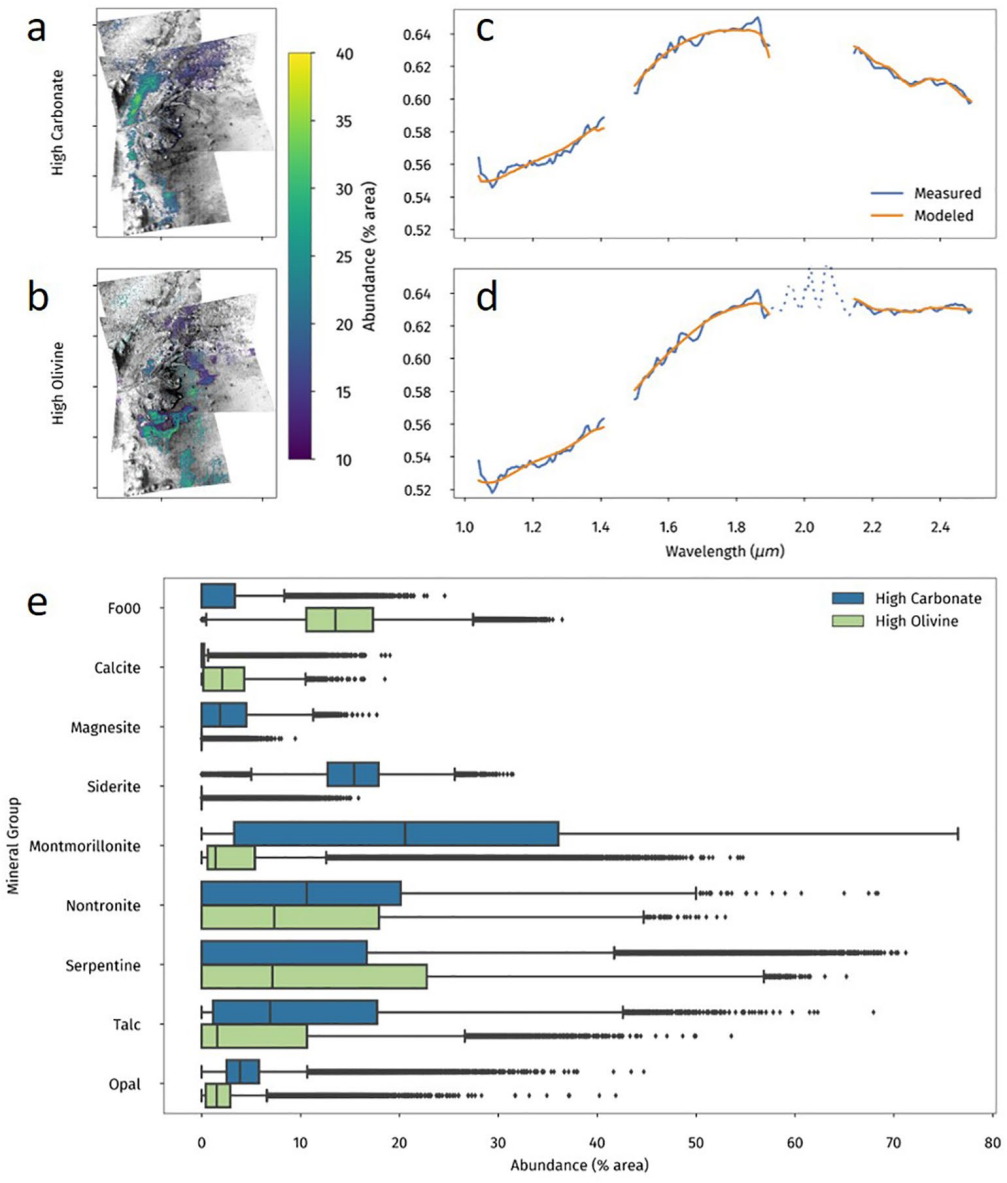
(a) Carbonate and (b) olivine abundances at their respective 90th percentiles and above. (c and d) Mean measured and modeled spectra for 90th percentile pixels. Mean spectra from all three CRISM images have been averaged together to produce a single spectrum. The dashed line on (d) from ∼1.9 to 2.1 µm represents residual atmosphere not removed by the DISORT model. A full explanation is in the supplementary material. (e) Distribution of abundances for all groups for both the high‐carbonate and high‐olivine regions. All distribution plots in this paper are standard box and whisker plots. A more detailed explanation is in the supplementary material. CRISM, Compact Reconnaissance Imaging Spectrometer for Mars.

We completed further analysis of the high‐carbonate and high‐olivine regions by isolating the pixels that contain abundances at and above the 90th percentile of the distribution for each mineral group. These masks and group distributions are shown in Figure 3. Abundance distributions for the specific minerals in each group are found in Figure S5.

**Figure 4:**
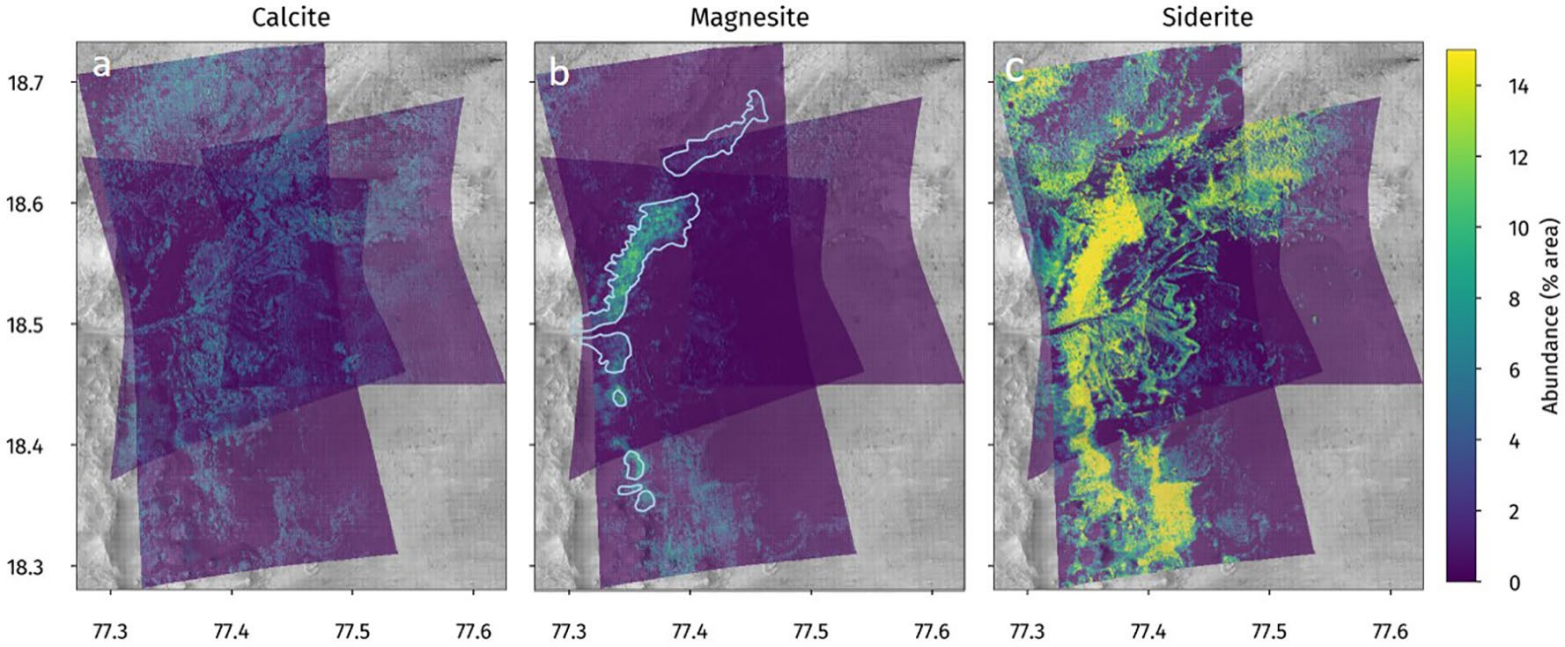
(a) Calcite, (b) magnesite, and (c) siderite abundance maps. The Marginal Carbonates are outlined again in (b).

The spatial distributions in Figure 3 show that the regions of high carbonate and olivine are inversely correlated. In terms of carbonate mineral distributions, the high‐carbonate regions are comprised mainly of magnesite and siderite and the high‐olivine regions are higher in calcite. Figure 4 shows the abundance maps for the three carbonates used in the model. Siderite is dominant and prevalent throughout the Marginal Carbonates and the Mottled Terrain. Magnesite is largely restricted to the Marginal Carbonates, which are outlined in the figure, although it is not altogether missing from the Mottled Terrain. Calcite appears in much lower abundances but is spread relatively evenly throughout the region, although it does appear to be anticorrelated with siderite.

**Figure 5:**
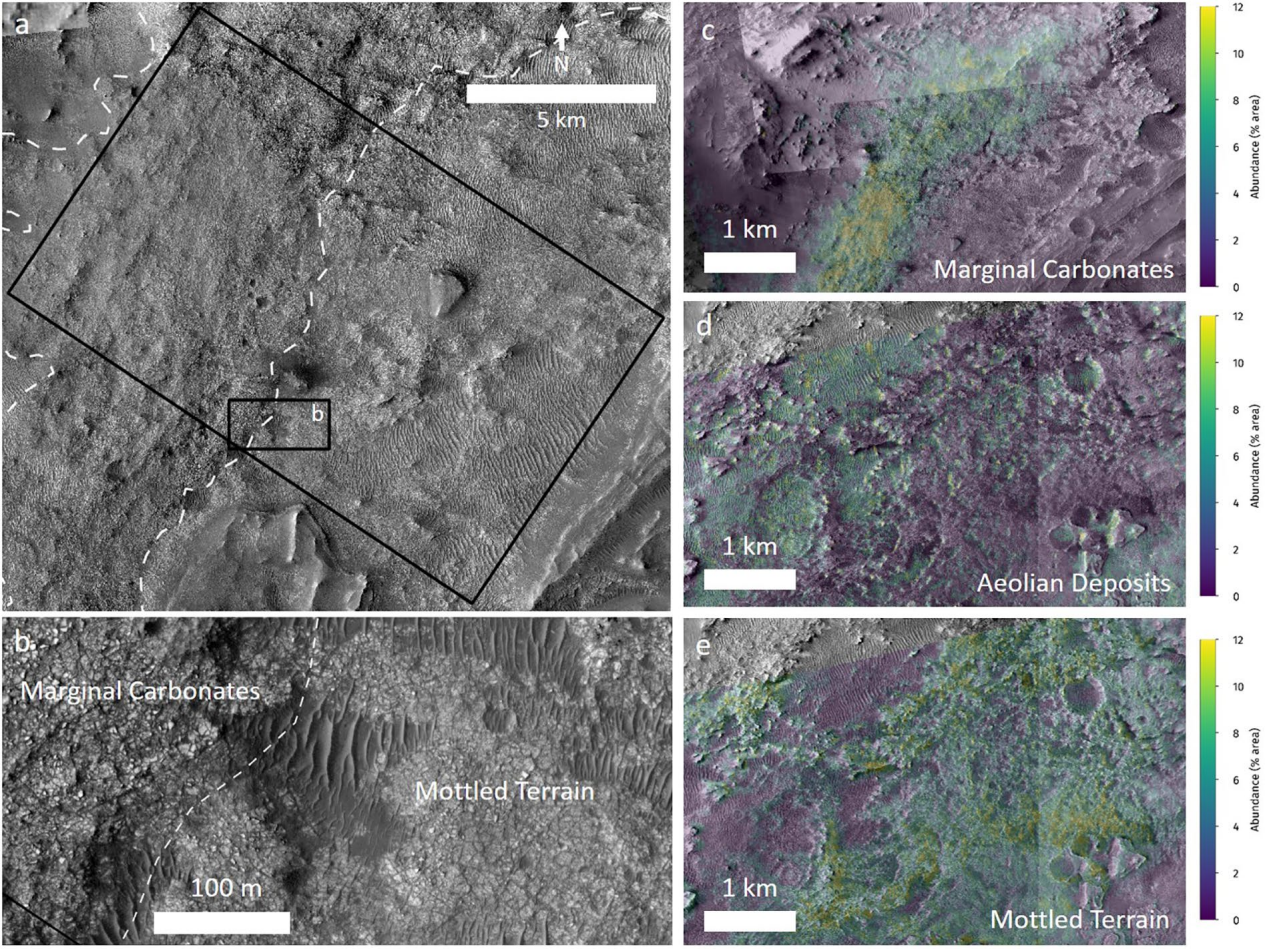
(a) Close‐up of ROI. (b) Zoom to highlight contact between Marginal Carbonates and Mottled Terrain. (c–e) Abundance maps of the three main carbonate units, context boxes in Figure 1. (c) Marginal Carbonates with magnesite abundances overlain, (d) olivine‐rich sand with calcite abundances overlain, and (e) Mottled Terrain with siderite abundances overlain. Base image is the Murray Lab HiRISE mosaic E77‐25_N18‐5.tif.

Finally, we mapped a region of interest (Figure 5a, 5b, and S6) that covers adjacent carbonate‐enriched and olivine‐enriched areas along the upper margin of the crater, as well as the potential contact between the Marginal Carbonates and the Mottled Terrain. Before mapping, the CRISM images were georegistered to the Murray Lab HiRISE mosaic. Units were mapped on the HiRISE image by CRISM pixel, i.e., each CRISM pixel was assigned to a unit based on the entire area covered by the pixel. We used HRL000040FF for this mapping because its RMS error is lower than the other image that overlaps this region. Each pixel was mapped as majority light‐toned, majority sand, or a mixture of light‐toned and sand. The majority light‐toned units were then split into the Marginal Carbonates and the Mottled Terrain by following the dashed line in Figure 5a. In the ROI, there are two other features that do not fall into any of these three categories. These areas have been masked out for the analysis. The mapping, mineral distributions, and statistical analysis are detailed in Figures S6 and S7 and summarized below.

Carbonate abundance is highest in the two light‐toned units and olivine abundance is highest in the sand unit and Mottled Terrain. Phyllosilicate abundance is equally high in both light‐toned units; however, the specific phyllosilicate mineral distributions show differences in spatial distribution. Talc is distributed throughout all three units; montmorillonite and nontronite are found least in the sand unit, and most in the Marginal Carbonates and Mottled Terrain, respectively; serpentine is found most in the sand unit, less in the Mottled Terrain, and not at all in the Marginal Carbonates; and saponite is not found in any of the units. Opal is found in similar abundances in the two light‐toned units and slightly more in the sand unit. In terms of carbonate phase, magnesite is found solely in the Marginal Carbonates, siderite is found in all three units, and calcite is found in the Mottled Terrain and sand units.

## 4 Discussion

### 4.1 The Nature of Olivine Deposits in Jezero

Based on our analysis, the olivine detected by CRISM in Jezero crater appears to be predominantly contained in the sand covering the terrain. These olivine‐rich sands likely are the result of preferential physical weathering of the host rock: in this case, the Light‐Toned Floor, consistent with Goudge et al. (2015). Visual examination of HiRISE images over the other high‐olivine areas shows they are almost exclusively sand‐dominated, particularly the deposits on the crater floor to the south and east of the delta. Examples of these areas are in Figure S7. Olivine is also present in the Mottled Terrain, but at abundances levels ∼4x less than in the sands.

Curiously, the dominant olivine endmember used by the baseline NNLS model is Fo_0_, with only Fo_70_ showing any other significant spatially resolved appearance (Figure S8). This is somewhat contrary to previous analyses of data from the Thermal Emission Spectrometer (TES, Koeppen & Hamilton, 2008) that have indicated that the olivine in this region is more Mg‐rich (∼Fo_50–75_) than our baseline model indicates. In addition, the Marginal Carbonates have long been considered to be olivine‐rich (e.g., Brown et al., 2020; Ehlmann et al., 2008; Goudge et al., 2015; Kremer et al., 2019; Mandon et al., 2020), which our model also somewhat disputes. These studies show no real distinction between the carbonate‐bearing and olivine‐bearing units as our preferred baseline model does. One of the findings of Brown et al. (2020) was a large grain size, Fo_44_–Fo_66_ olivine composition. It is possible that, having reached the upper limits of grain size (1,000 µm) with olivine in our model, the model has compensated by using a more Fe‐rich olivine.

To further test our model's preference for siderite over magnesite, calcite, and the more Mg‐rich olivine endmembers, we included a model run with the Fe‐carbonate endmember removed. This model results in higher abundances of more Mg‐rich olivine in the Marginal Carbonates. Maps showing the change in abundance for Fo_40_ and Fo_70_ in HRL000040FF are in Figure S3a.

In terms of quality of spectral fit, however, the inclusion of siderite is clearly preferred by our model. The RMS error is smaller for the baseline model and the spectra match more closely, particularly around ∼2.3 µm, where siderite has an absorption band. Modeled spectra from both models of the high‐carbonate region, as identified in Figure 3, are shown in Figure S3b. Horgan et al. (2020) noted that the spectral shape in the Marginal Carbonates was probably due to Fe‐substitution in carbonate as opposed to Fe‐substitution in other minerals. Our model appears to corroborate that suggestion.

Taking into account both our model results and those of past analyses (e.g., Goudge et al., 2015; Horgan et al., 2020), we consider it likely that the unit's composition is somewhere in between our two best‐fit models with a mixture of olivine and more Fe‐bearing Mg‐carbonate in the Marginal Carbonates.

### 4.2 Carbonate Variability

Horgan et al. (2020) noted that the spectra in the Marginal Carbonates differed from those in the Mottled Terrain in both relative strength and the ratio of the carbonate absorptions at 2.3  and 2.5 μm. This difference is mostly likely due to differences in formation processes and environment. Their preferred method for the Marginal Carbonate's formation is a combination of fluvial activity and precipitation in a near‐shore lacustrine environment.

Our model supports the hypothesis that there is a difference between the carbonates identified in the Marginal Carbonates and in the Mottled Terrain based on changing carbonate mineralogy in the two areas. To summarize the results shown above, siderite is present in both the Marginal Carbonates and the Mottled Terrain, magnesite is almost entirely found in the Marginal Carbonates, and calcite is more associated with the sandy regions (greater Fo_0_ abundance) than either of the more‐lithified terrains.

From this analysis, there appear to be three distinct carbonate‐bearing units across the study region. The most prominent unit is the Marginal Carbonates, made up of siderite and magnesite (Figure 5c). The second carbonate‐bearing unit includes calcite associated with the olivine‐rich sands largely found around the Mottled Terrain (Figure 5d). The third unit is the Mottled Terrain with siderite in the more‐lithified landforms (Figure 5e).

### 4.3 Uncertainty in Phyllosilicate Modeling

The distribution of phyllosilicate minerals, as well as the selection of particular minerals, is cause for some concern in analyzing our modeling results. In regions such as the crater floor and the high‐olivine units, where the spectra from 2.1 to 2.5 µm are generally flat, the phyllosilicate values tend to be quite high. A possible explanation for this is that the model is trying to use the phyllosilicate spectra (which have sharp features) to model the noise in the spectra instead of the flat “features”. In terms of phyllosilicate mineral selection, it is confusing that the model has chosen an Al‐smectite (montmorillonite) over the previously identified Fe/Mg‐smectites (saponite). However, the uncertainty in the phyllosilicate abundances does not appear to greatly affect the other mineral groups. We ran a series of models removing each of the phyllosilicate minerals and opal and compared them to the base model. Overall, when the phyllosilicates were removed, the only phase that was largely affected (i.e., by more than a couple of percent) was dust, which increased greatly when endmembers were removed. Figure S9 shows these results.

### 4.4 Implications for Carbonate Formation and Jezero History

Our analysis identifies three carbonate assemblages in Jezero crater, indicating that there were likely at least three potential periods of carbonate formation. Below, we outline and discuss these periods and hypothesize a potential formation sequence.

#### 4.4.1 Olivine Deposition and Calcite Formation

A number of hypotheses for the formation of the olivine‐rich unit in Jezero and the wider Nili Fossae region have been suggested. The hypothesis that the olivine was deposited as volcanic ash has been supported by a number of researchers (e.g., Kremer et al., 2019; Mandon et al., 2020; Rogers et al., 2018). Horgan et al. (2020) noted a potential terrestrial analogue in the Igwisi Hills lavas and tuffs that contain primary olivine, secondary calcite, and a serpentine‐like mineral (Willcox et al., 2015). The ash hypothesis is supported by the presence of calcite mixed with the olivine‐rich sands here in Jezero as identified by our model.

Initial partial serpentinization and formation of calcite in the olivine‐bearing unit may have occurred rapidly after emplacement due to contact between the hot ash and the water‐rich layer beneath it (e.g., Brown et al., 2010; Ehlmann et al., 2008; Viviano et al., 2013). Mandon et al. (2020) questioned this hypothesis due to the presence of carbonate across many tens of meters of elevation in the same unit, whereas contact metamorphism would only occur in the first meters of the unit. However, it is not necessary that all of the carbonate in Jezero formed directly after the ash was emplaced.

#### 4.4.2 Initial Fe‐Rich Carbonate Formation

We hypothesize that at some point after the ash emplacement and partial serpentinization (or perhaps concurrently, as suggested by Brown et al. (2010)), partial carbonation of serpentine occurred. The preference of our model for more Fe‐rich olivine is consistent with a resulting carbonate that is also more Fe‐rich. Given the sharp decrease of olivine in the Mottled Terrain as compared to the sand unit, perhaps serpentinization occurred more fully at the top of the unit (as suggested by van Berk and Fu (2011)), leaving the olivine‐rich ash, calcite, and serpentine that we see most strongly in the sand unit to later be broken down by physical weathering.

#### 4.4.3 Marginal Carbonate Formation

Given the modeled occurrence of siderite in both the Mottled Terrain and the Marginal Carbonates, it is possible that both units underwent the same emplacement and initial alteration to serpentine and Fe‐rich carbonate. Thus, the production of more Mg‐rich carbonate now present in the Marginal Carbonates must have occurred at some time after the initial alteration. Horgan et al. (2020) hypothesized that these carbonates formed as a combination of fluvial and beach deposits in a different environment than the Mottled Terrain. The change in carbonate phase from Fe‐rich to Mg‐rich that our model has detected is a potential indicator of formation in different environments; thus, we agree that the formation of Mg‐carbonate as a lake‐side precipitate is possible. The lack of olivine and serpentine in the Marginal Carbonates does indicate that additional serpentinization and carbonation occurred here as well.

## 5 Conclusions

Our analysis has identified three carbonate‐bearing units with different carbonate phase representations: (1) the Marginal Carbonates with high Mg‐rich and Fe‐rich carbonate and a lack of olivine and serpentine; (2) the Mottled Terrain with Fe‐rich carbonate and less significant amounts of olivine of serpentine; and (3) the olivine‐rich sands enriched in finely particulate calcite and serpentine. Total carbonate abundances across the study area reach up to 35% per pixel. The Marginal Carbonates have the highest overall carbonate abundance with 50% of the abundances falling between ∼15% and 20% abundance, followed by the Mottled Terrain with 50% of the abundances falling between ∼10% and 15%, and lastly by the olivine‐rich sands with 50% of the abundances falling between 5% and 8%. The error analysis presented in Figure S4 confirms that these abundances are likely detectable by our model.

The diversity of carbonate phases present in Jezero points to multiple periods of carbonate formation under different circumstances. We posit that (1) olivine‐rich ash was deposited across Jezero, partially serpentinized and altered to form calcite, (2) subsequently altered to an Fe‐rich carbonate, and (3) in the case of the Marginal Carbonates, underwent another period of alteration to or were precipitated as a more Mg‐rich carbonate. In‐situ analysis and sample collection by the Mars 2020 rover will be crucial to understanding the full extent of carbonate formation in Jezero crater.

## Supporting information

Supporting Information S1

## Data Availability

Original CRISM data sets are from the Planetary Data System's Geosciences Node (https://pds-geosciences.wustl.edu/). Processed and modeled data may be found in Zastrow and Glotch (2020) (https://doi.org/10.5281/zenodo.4699583). CTX and HiRISE mosaics are from The Murray Lab at the California Institute of Technology (http://murray-lab.caltech.edu/Mars2020/index.html).

## References

[grl62287-bib-0001] Brown, A. J. , Hook, S. J. , Baldridge, A. M. , Crowley, J. K. , Bridges, N. T. , Thomson, B. J. , et al. (2010). Hydrothermal formation of clay‐carbonate alteration assemblages in the Nili Fossae region of Mars. Earth and Planetary Science Letters, 297(1–2), 174–182. 10.1016/j.epsl.2010.06.018

[grl62287-bib-0002] Brown, A. J. , Viviano, C. E. , & Goudge, T. A. (2020). Olivine‐carbonate mineralogy of the Jezero crater region. Journal of Geophysical Research: Planets, 125(3), e2019JE006011. 10.1029/2019JE006011 PMC7592698 33123452

[grl62287-bib-0003] Edwards, C. S. , & Ehlmann, B. L. (2015). Carbon sequestration on Mars. Geology, 43(10), 863–866. 10.1130/G36983.1

[grl62287-bib-0004] Ehlmann, B. L. , Mustard, J. F. , Murchie, S. L. , Poulet, F. , Bishop, J. L. , Brown, A. J. , et al. (2008). Orbital identification of carbonate‐bearing rocks on Mars. Science, 322(5909), 1828–1832. 10.1126/science.1164759 19095939

[grl62287-bib-0005] Farley, K. A. , Williford, K. H. , Stack, K. M. , Bhartia, R. , Chen, A. , de la Torre, M. , et al. (2020). Mars 2020 mission overview. Space Science Reviews, 216(8), 142. 10.1007/s11214-020-00762-y

[grl62287-bib-0006] Goudge, T. A. , Mustard, J. F. , Head, J. W. , Fassett, C. I. , & Wiseman, S. M. (2015). Assessing the mineralogy of the watershed and fan deposits of the Jezero crater paleolake system, Mars. Journal of Geophysical Research: Planets, 120, 775–808. 10.1002/2014JE004782

[grl62287-bib-0007] Horgan, B. H. N. , Anderson, R. B. , Dromart, G. , Amador, E. S. , & Rice, M. S. (2020). The mineral diversity of Jezero crater: Evidence for possible lacustrine carbonates on Mars. Icarus, 339, 113526. 10.1016/j.icarus.2019.113526

[grl62287-bib-0008] Itoh, Y. , & Parente, M. (2021). A new method for atmospheric correction and de‐noising of CRISM hyperspectral data. Icarus, 354, 114024. 10.1016/j.icarus.2020.114024

[grl62287-bib-0009] Koeppen, W. C. , & Hamilton, V. E. (2008). Global distribution, composition, and abundance of olivine on the surface of Mars from thermal infrared data. Journal of Geophysical Research, 113, E05001. 10.1029/2007JE002984

[grl62287-bib-0010] Kremer, C. H. , Mustard, J. F. , & Bramble, M. S. (2019). A widespread olivine‐rich ash deposit on Mars. Geology, 47(7), 677–681. 10.1130/G45563.1

[grl62287-bib-0011] Lapotre, M. G. A. , Ehlmann, B. L. , & Minson, S. E. (2017). A probabilistic approach to remote compositional analysis of planetary surfaces. Journal of Geophysical Research: Planets, 122, 983–1009. 10.1002/2016JE005248

[grl62287-bib-0012] Lawson, C. L. , & Hanson, R. J. (1995). Solving Least Squares Problems. Philadelphia, PA: SIAM. 10.1137/1.9781611971217

[grl62287-bib-0013] Liu, Y. , Arvidson, R. E. , Wolff, M. J. , Mellon, M. T. , Catalano, J. G. , Wang, A. , & Bishop, J. L. (2012). Lambert albedo retrieval and analyses over Aram Chaos from OMEGA hyperspectral imaging data: Lambert albedo retrieval for Aram Chaos. Journal of Geophysical Research, 117, E00J11. 10.1029/2012JE004056

[grl62287-bib-0014] Liu, Y. , Glotch, T. D. , Scudder, N. A. , Kraner, M. L. , Condus, T. , Arvidson, R. E. , et al. (2016). End‐member identification and spectral mixture analysis of CRISM hyperspectral data: A case study on southwest Melas Chasma, Mars. Journal of Geophysical Research: Planets, 121, 2004–2036. 10.1002/2016JE005028

[grl62287-bib-0015] Mandon, L. , Quantin‐Nataf, C. , Thollot, P. , Mangold, N. , Lozac'h, L. , Dromart, G. , et al. (2020). Refining the age, emplacement and alteration scenarios of the olivine‐rich unit in the Nili Fossae region, Mars. Icarus, 336, 113436. 10.1016/j.icarus.2019.113436

[grl62287-bib-0016] Michalski, J. R. , Glotch, T. D. , Rogers, A. D. , Niles, P. B. , Cuadros, J. , Ashley, J. W. , & Johnson, S. S. (2019). The geology and astrobiology of McLaughlin Crater, Mars: An ancient lacustrine basin containing turbidites, mudstones and serpentinites. Journal of Geophysical Research: Planets, 124(4), 910–940. 10.1029/2018JE005796

[grl62287-bib-0017] Murchie, S. , Arvidson, R. , Bedini, P. , Beisser, K. , Bibring, J.‐P. , Bishop, J. , et al. (2007). Compact reconnaissance imaging spectrometer for Mars (CRISM) on Mars reconnaissance orbiter (MRO). Journal of Geophysical Research, 112, E05S03. 10.1029/2006JE002682

[grl62287-bib-0018] Rogers, A. D. , & Aharonson, O. (2008). Mineralogical composition of sands in Meridiani Planum determined from Mars Exploration Rover data and comparison to orbital measurements. Journal of Geophysical Research: Planets, 113, E06S14. 10.1029/2007JE002995

[grl62287-bib-0019] Rogers, A. D. , Warner, N. H. , Golombek, M. P. , Head, J. W. , & Cowart, J. C. (2018). Areally extensive surface bedrock exposures on Mars: Many are clastic rocks, not lavas. Geophysical Research Letters, 45, 1767–1777. 10.1002/2018GL077030 30598561 PMC6310033

[grl62287-bib-0020] Salvatore, M. R. , Goudge, T. A. , Bramble, M. S. , Edwards, C. S. , Bandfield, J. L. , Amador, E. S. , et al. (2018). Bulk mineralogy of the NE Syrtis and Jezero crater regions of Mars derived through thermal infrared spectral analyses. Icarus, 301, 76–96. 10.1016/j.icarus.2017.09.019

[grl62287-bib-0021] van Berk, W. , & Fu, Y. (2011). Reproducing hydrogeochemical conditions triggering the formation of carbonate and phyllosilicate alteration mineral assemblages on Mars (Nili Fossae region). Journal of Geophysical Research, 116, E10006. 10.1029/2011JE003886

[grl62287-bib-0022] Viviano, C. E. , Moersch, J. E. , & McSween, H. Y. (2013). Implications for early hydrothermal environments on Mars through the spectral evidence for carbonation and chloritization reactions in the Nili Fossae region. Journal of Geophysical Research: Planets, 118, 1858–1872. 10.1002/jgre.20141

[grl62287-bib-0023] Willcox, A. , Buisman, I. , Sparks, R. S. J. , Brown, R. J. , Manya, S. , Schumacher, J. C. , & Tuffen, H. (2015). Petrology, geochemistry and low‐temperature alteration of lavas and pyroclastic rocks of the kimberlitic Igwisi Hills volcanoes, Tanzania. Chemical Geology, 405, 82–101. 10.1016/j.chemgeo.2015.04.012

[grl62287-bib-0024] Williford, K. H. , Farley, K. A. , Stack, K. M. , Allwood, A. C. , Beaty, D. , Beegle, L. W. , et al. (2018). The NASA Mars 2020 rover mission and the search for extraterrestrial life. In N. A. Cabrol , & E. A. Grin (Eds.), From habitability to life on Mars (pp. 275–308). Amsterdam, Netherlands: Elsevier. 10.1016/B978-0-12-809935-3.00010-4

[grl62287-bib-0025] Wolff, M. J. , Smith, M. D. , Clancy, R. T. , Arvidson, R. , Kahre, M. , Seelos, F. , et al. (2009). Wavelength dependence of dust aerosol single scattering albedo as observed by the compact reconnaissance imaging spectrometer. Journal of Geophysical Research, 114, E00D04. 10.1029/2009JE003350

[grl62287-bib-0027] Zastrow, A. M. , & Glotch, T. D. (2018). CRISM atmospheric correction with DISORT: Variation of pressure and opacity input parameters on derived single scattering albedo and linear unmixing. AGU Fall Meeting Abstracts, 53. http://adsabs.harvard.edu/abs/2018AGUFM.P53F3027Z

[grl62287-bib-0026] Zastrow, A. M. , & Glotch, T. D. (2020). Data and code used in “Distinct Lithologies in Jezero Crater, Mars” (Version 1.2.0) [Data set]. Zenodo. 10.5281/zenodo.4408577 PMC8243932 34219844

