# Supplementary material for: Distinct Carbonate Lithologies in Jezero Crater, Mars
Source: Geophys Res Lett. 2021 May 6;48(9):e2020GL092365. doi: 10.1029/2020GL092365 (PMC8243932; doi:10.1029/2020GL092365)
Supplement: Supplementary file 1 — Supporting Information S1 [file GRL-48-e2020GL092365-s001.pdf]

AGU PUBLICATIONS

Geophysical Research Letters

Supporting Information for

Distinct Carbonate Lithologies in Jezero Crater, Mars

Allison M. Zastrow[1], Timothy D. Glotch[1]

[1]Stony Brook University, Stony Brook, NY, USA.

Contents of this file

Text S1 to S3
Figures S1 to S9
Tables S1 to S2

Introduction

This document contains further text describing our selection of the final model presented in this paper, as well as additional figures and tables that lend further detail to the description of the study area.

Text S1. Description of DISORT Atmospheric Correction

The model uses the Discrete Ordinate Radiative Transfer (DISORT) code (Stamnes et al., 1988), which is a one-dimensional radiative transfer model that separates the atmosphere from the surface. The atmosphere is treated as a series of layers that each account for a different atmospheric parameter. We include dust and ice aerosol optical depths (derived from CRISM and MRO Mars Color Imager (MARCI) data, Wolff et al., 2007), surface pressure (determined by using CO_2 abundance derived from CRISM emission phase function (EPF) measurements, Smith et al., 2009), and water vapor and temperature/pressure profiles (based on Mars Global Surveyor (MGS) Thermal Emission Spectrometer (TES) data at the appropriate latitude, longitude, and season, (Smith, 2004)).

The surface is modeled as the bottom layer using the Hapke bidirectional reflectance function (Hapke, 1981, 1993, 2012). To quantify mineralogy, we utilize the minerals' wavelength-dependent optical constants n and k to model CRISM data in terms of bulk single-scattering albedo (SSA, ω), which depends on n, k, and the grain size. The Hapke model was designed to approximate the effects of multiple scattering within a material before light is reflected out of the surface. The approximation of these effects results in linear mixing of SSAs.

To atmospherically correct a CRISM image, the surface scattering parameters, atmospheric parameters, and viewing geometry (from the CRISM image itself) are input into the Hapke multiple scattering function to output lookup tables of single scattering albedo for each wavelength, which then replace the corresponding I/F values in the CRISM image. Surface pressure is varied until residual atmospheric effects are minimized and the final surface SSA output is produced.

Text S2. Spectral Library and Best-Fit Model Selection

After testing our unmixing model on multiple spectral ranges, we cut out regions of the spectra between ~1.4-1.5 µm and ~1.9-2.1 µm to reduce spectral noise due to residual features from the atmospheric correction. This greatly improved the model errors and removed column-correlated noise from the residual root-mean-squared (RMS) error images, as shown in Figure S1. Some striping in the RMS error image still remains, but the overall error values are significantly smaller in the cut spectra and the difference in the error values of the stripes is also much smaller. An additional cut from ~1.6-1.7 reduces the error striping further; however, the model results do not change appreciably, so we have chosen to not use this cut to avoid losing any more spectral data. Removing these portions of the spectra will likely affect the derived abundances of minerals with water in their structures. Therefore, abundances of minerals like phyllosilicates should be regarded as having more uncertainty.

Our spectral library was originally modeled after the library used in Michalski et al. (2019). We removed the carbonate minerals of ankerite and dolomite to focus on the three main endmembers. We removed a couple of phyllosilicate minerals such as

vermiculite due to erroneous occurrences like with kaolinite outlined below. Lastly, we added the serpentine and talc endmembers to test the hypothesis that serpentinization was an important process in Jezero.

Additionally, we adjusted the model to in a couple of ways to improve the match between the three images. The three modeled images show good agreement in overlapping areas, particularly north of the crater’s inlet channel (where most of our analysis is focused). We adjusted the grain sizes used for the phyllosilicates so that those abundances matched between the three images. Olivine is slightly higher in HRL000040FF than the other two images, but the other groups have very similar values. We also adjusted the grain sizes of the feldspar minerals (which are spectrally featureless in the range of our model) to better match feldspar abundances derived from TES data and they are consistent (considering the difference in spatial resolution).

For each CRISM image, we ran a series of models with various combinations of endmember minerals and compared their RMS errors and modeled spectra. Here, we briefly outline our reasons for selecting the model presented in this paper. We present three of the models from which we made our selection: 1) with slope parameters added, 2) with kaolinite added, and 3) with siderite removed.

The first model added two slope parameters to the spectral library (essentially straight lines from 0 to 1 and from 1 to 0) across the spectral region. The purpose in adding these slope parameters is to potentially correct for any systematic sloping in the CRISM images. In our case we found that the slope “abundance” maps follow closely to geologic landforms; thus, we did not choose to use this model.

The second model added a set of kaolinite endmembers to the spectral library. The inclusion of kaolinite into the model did result in a decrease in RMS error (from 0.00213 to 0.00200 calculated between 2.1 and 2.5 um); however, the resulting modeled spectra of the high-carbonate regions are not a better match as seen in Figure S2 at ~2.2 um. Thus, we chose to not use this model as well.

The third model removed the siderite endmembers from the spectral library. In this case, there was an increase in RMS error when siderite was removed (from 0.00213 to 0.00243 calculated between 2.1 and 2.5 um, the largest change in RMS error when a mineral was removed from the library) and the modeled spectra with siderite removed deviate more from the measured spectra, particularly around ~2.3 um. Because of this, we chose not to use this model.

In testing the other phyllosilicate minerals as above, we determined that minerals such as montmorillonite should be left in the model (despite not being identified previously), because the match of measured and modeled spectra in the high-carbonate regions improved when these minerals were present.

Text S3. Box and Whisker Plots

In this paper, we use box and whisker plots to plot and compare the distribution of mineral and cumulative mineral group abundances in different image pixels. The box covers the interquartile range (IQR) and is created by calculating the 25th, 50th, and 75th percentiles for each mineral/group, and these form the left end, center line, and right end of the box, respectively. The whiskers extend to (25th percentile value – 1.5 * IQR) on the left and (75th percentile value + 1.5 * IQR) on the right. The points are statistical outliers beyond the whiskers. When analyzing these plots, it is important to note that minerals/groups that have abundance values that are overwhelmingly equal to 0, but have some pixels with higher abundance values, will have more of these outlier points and little to no box.

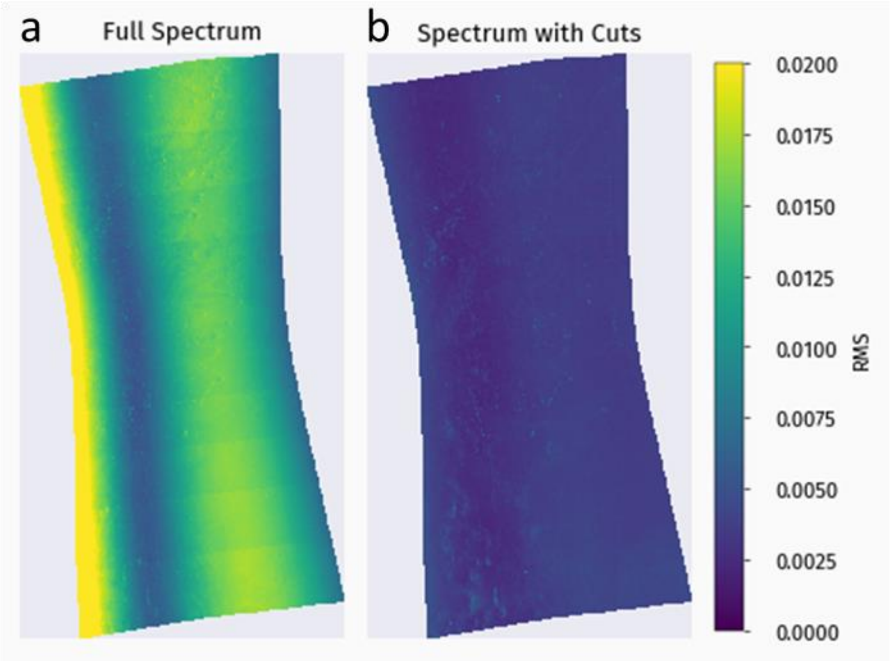

Figure S1. Change in model RMS error for HRL040FF a) over the full spectral range and b) with two cuts between ~1.4-1.5 µm and ~1.9-2.1 µm.

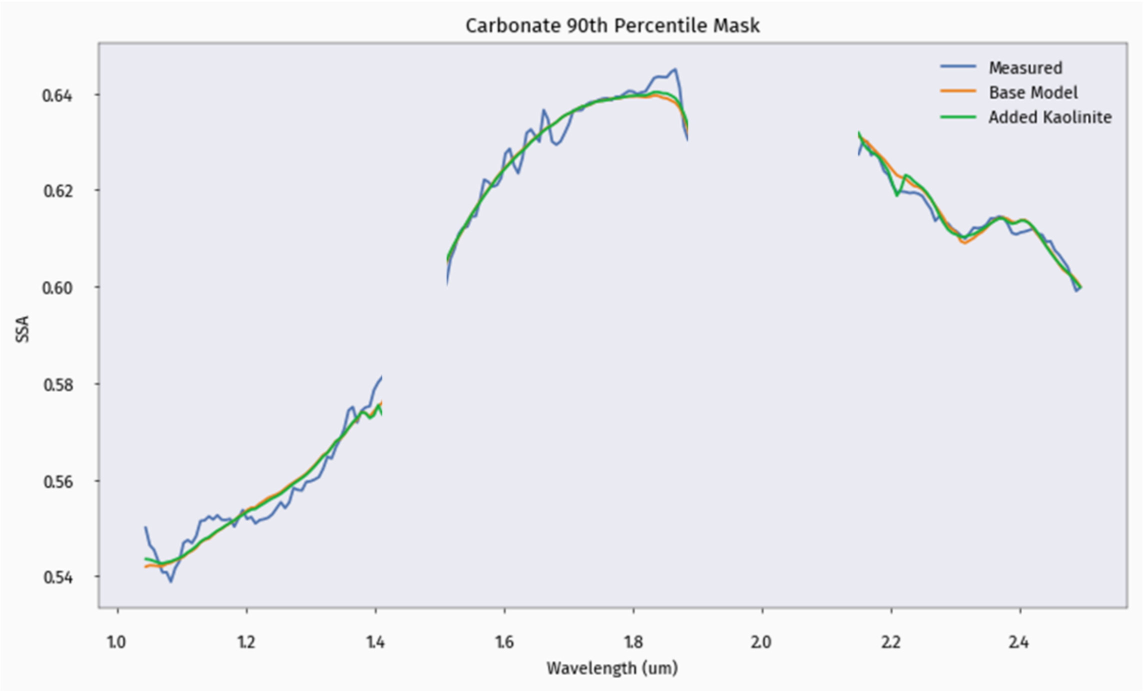

Figure S2. Mean spectra comparison for model with and without kaolinite endmembers.

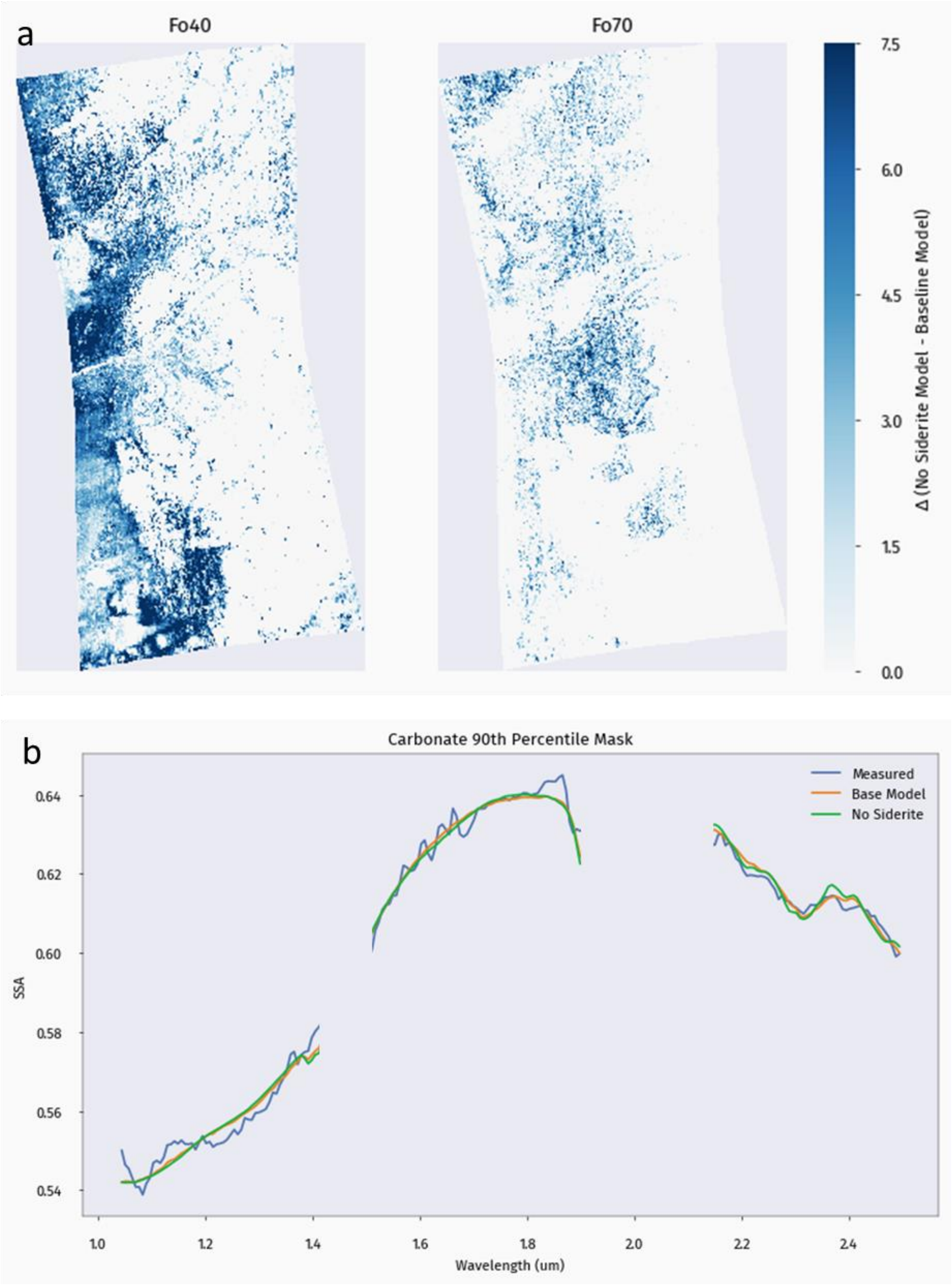

Figure S3. a) Change in olivine abundance maps modeled without siderite and b) mean spectra comparison. Excluding siderite from the model increases Fo40 and Fo70 abundances by up to ~7.5%.

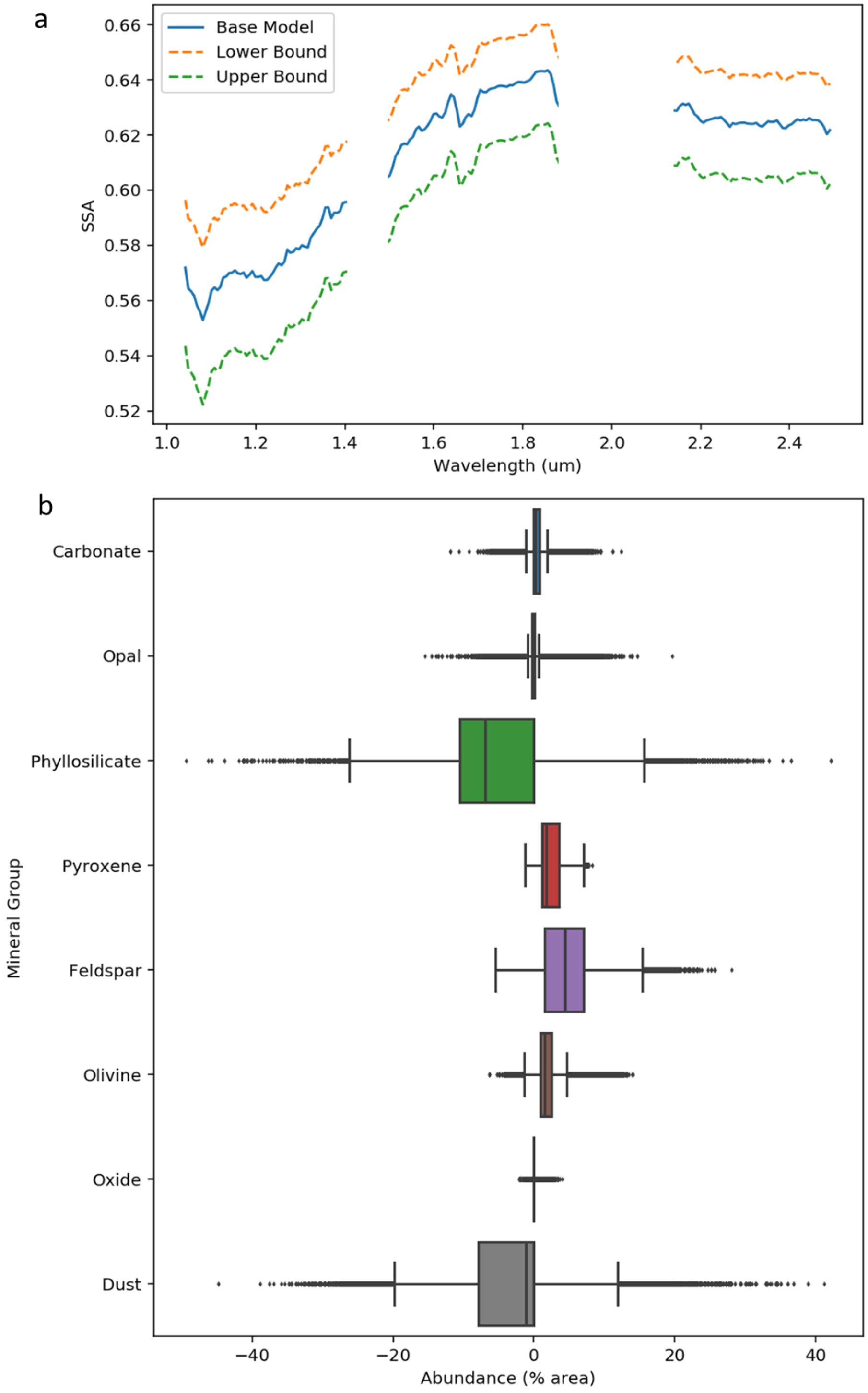

Figure S4. a) Average spectra for the base model, upper, and lower atmospheric bounds for image HRL000047A3 and b) Distribution for each mineral group representing the difference in modeled abundance between for the upper and lower bound models.

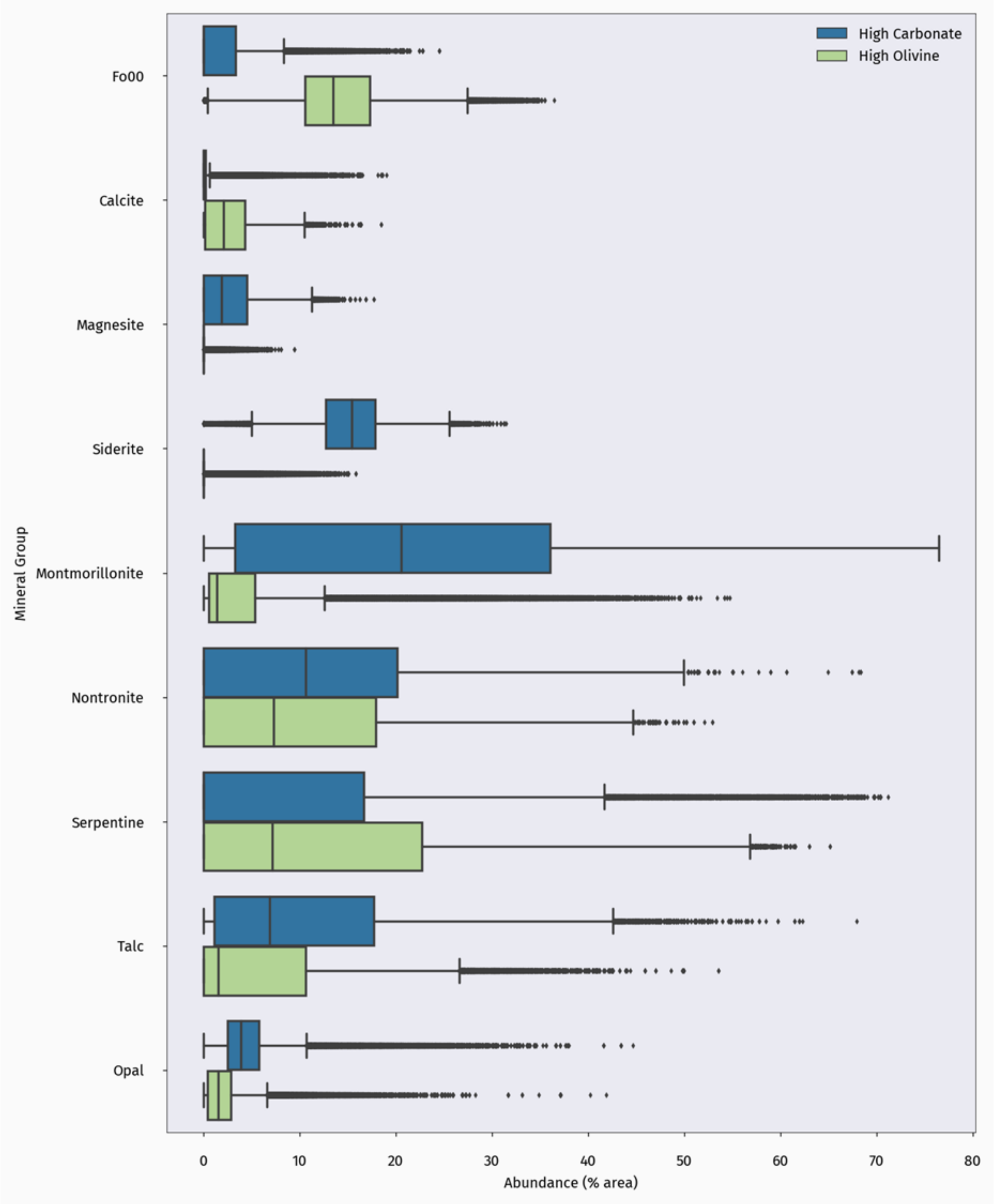

Figure S5. Modeled abundances for specific minerals in the carbonate, phyllosilicate, olivine, and opal groups for the high-carbonate and high-olivine regions mapped in Figure 3.

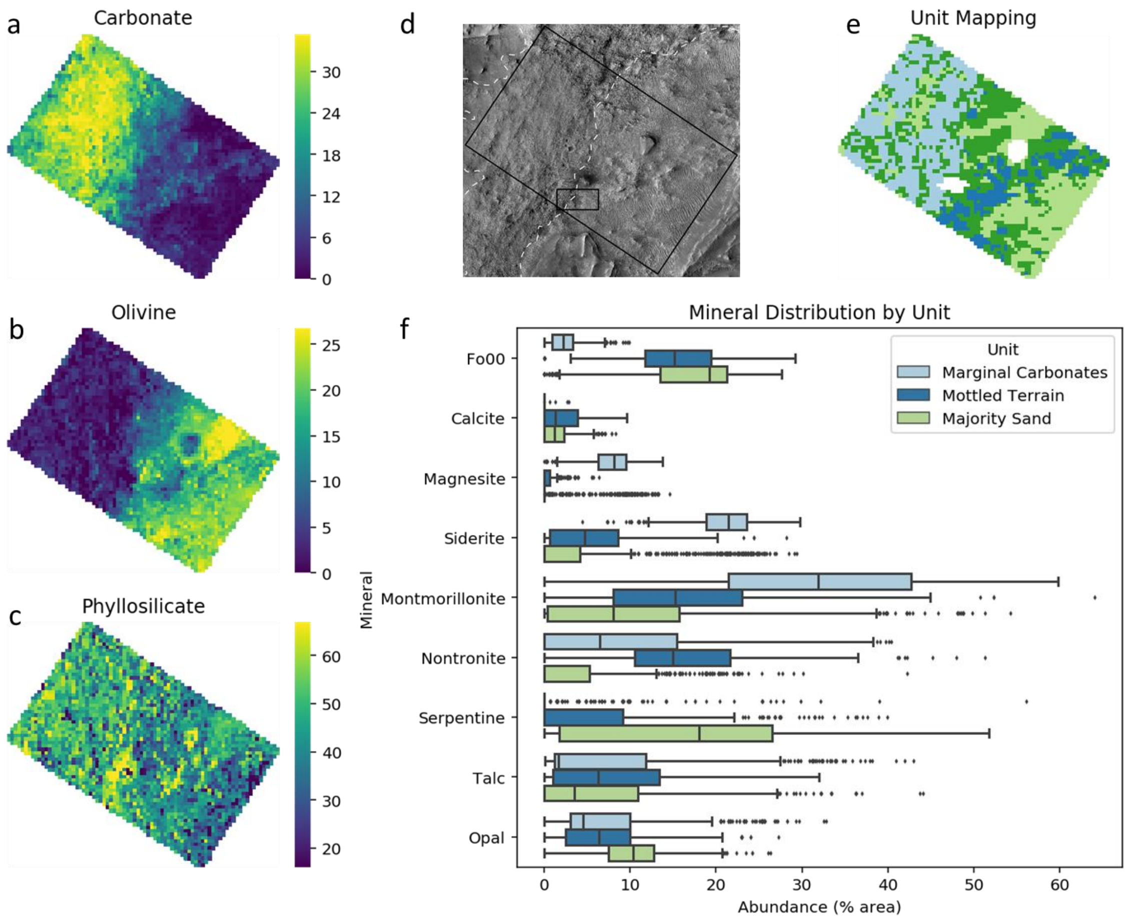

Figure S6. Detailed mineral/group distribution in the ROI. Modeled abundances for a) carbonate, b) olivine, and c) phyllosilicate; d) HiRISE image (duplicate from Figure 5a); e) unit mapping, colors match the units in part f, dark green pixels are the "mixture" pixels, which we have not included in the distribution mapping; f) Mineral distribution by unit. Results are discussed in the main article.

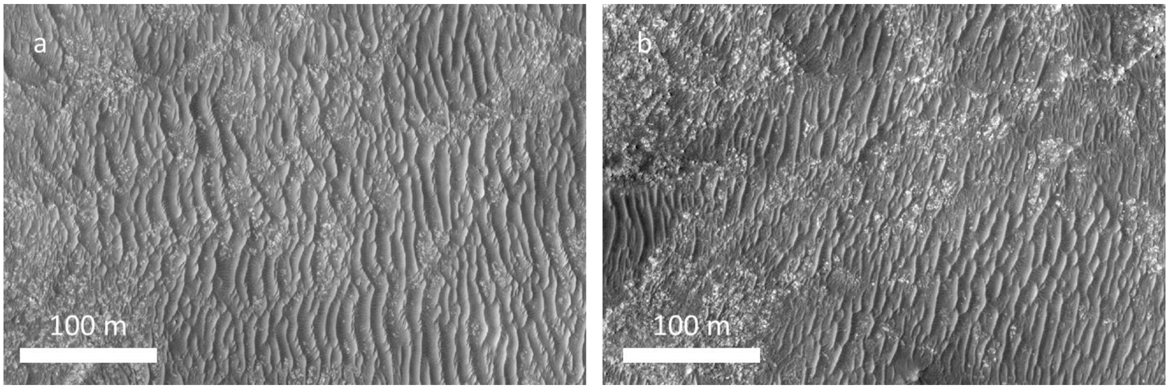

Figure S7. Examples of olivine-rich areas (light-toned floor unit from Goudge et al. (2015)). Images are from the Murray Lab HiRISE Mosaic.

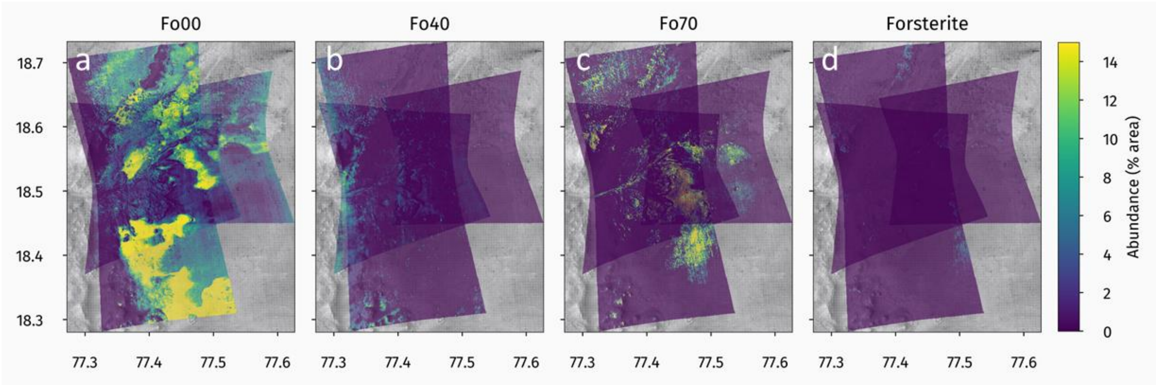

Figure S8. Breakdown of olivine endmembers. a) Fo_0, b) Fo_{40}, c)Fo_{70}, and d) Fo_{100} or Forsterite.

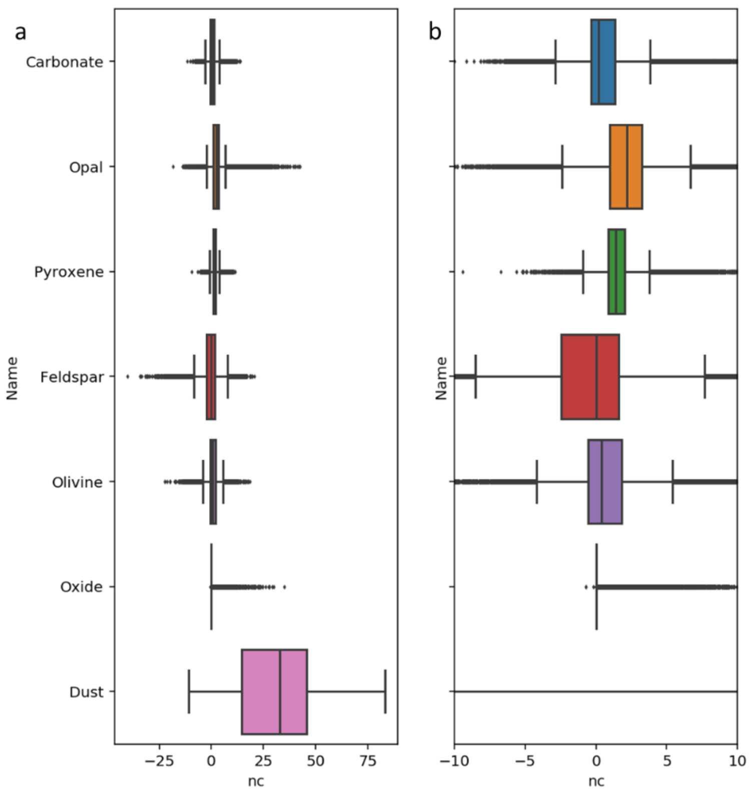

Figure S9. Difference between the base model and the model with all phyllosilicates removed. Positive values indicate that the abundances increased when all phyllosilicates were removed, negative values indicate the abundances decreased. a) covers the full range and b) is zoomed in to -10 to +10% difference.

**Image ID**	τ_d	τ_i	**Resolution (m/pix)**
HRL040FF	0.45 ± 0.05	0.04 ± 0.03	36
FRT047A3	0.53 ± 0.07	0.05 ± 0.03	18
FRT05C5E	0.93 ± 0.10	0.03 ± 0.03	18

Table S1. Dust and ice opacity values and image resolutions for the three CRISM images analyzed in this work.

**Mineral**	**Grain Sizes (μm)**
Calcite	50, 150, 400, 800
Magnesite	
Siderite	
Opal	
Montmorillonite	5, 50, 250, 400, 800
Nontronite	
Saponite	
Mg-Serpentine	
Talc	
Ferrihydrite	1, 5, 15
Dust	
Fayalite	100, 400, 700, 1000
$Forsterite_{40}$	
$Forsterite_{70}$	
Forsterite	
Augite	
Diopside	
$Enstatite_{75}$[a]	
$Enstatite_{85}$[a]	
Anorthite	
Labradorite	

Table S2. Mineral grain sizes used in the model. Unless otherwise noted, optical constants were derived using the model from Shkuratov et al. (1999). [a]Derived using a Hapke model.

	Marginal Carbonates to Mottled Terrain	Marginal Carbonates to Majority Sand	Mottled Terrain To Majority Sand
**Critical Value**	**0.132**	**0.101**	**0.133**
Carbonate	0.926	0.807	0.543
Olivine	0.919	0.803	0.305
Phyllosilicate	0.128	0.465	0.414
Calcite	0.629	0.720	0.200
Magnesite	0.934	0.798	0.150
Siderite	0.889	0.791	0.478
Montmorillonite	0.469	0.644	0.267
Nontronite	0.378	0.297	0.656
Serpentine	0.348	0.701	0.432
Talc	0.211	0.292	0.254
Opal	0.152	0.441	0.342

Table S3. Results of a Kolmogorov-Smirnov statistical test (Hodges, 1958) to determine whether two mineral/group distributions come from the same distribution or not. Each pair of units has its own critical value, which is based on the number of pixels being compared, as well as the level of sensitivity. We used a high sensitivity in this test (α=0.001). The closer a K-S value is to the critical value, the more likely it is that two distributions are from the same distribution (in our physical case, this means that there is roughly the same amount of a mineral/group in both units). The closer a value is to 1, the more likely it is that they are not from the same distribution (physically, this means the abundances are not the same in both units). We utilized the K-S test for two reasons: 1) it does not assume the distributions are normal and 2) it is more appropriate for larger sample sizes.
